# Radiation-induced rib fracture after stereotactic body radiotherapy with a total dose of 54–56 Gy given in 9–7 fractions for patients with peripheral lung tumor: impact of maximum dose and fraction size

**DOI:** 10.1186/s13014-015-0406-8

**Published:** 2015-04-22

**Authors:** Masahiko Aoki, Mariko Sato, Katsumi Hirose, Hiroyoshi Akimoto, Hideo Kawaguchi, Yoshiomi Hatayama, Shuichi Ono, Yoshihiro Takai

**Affiliations:** Department of Radiology and Radiation Oncology, Graduate School of Medicine, Hirosaki University, 5 Zaifu-cho, Hirosaki, Aomori 036-8562 Japan; Department of Radiation Oncology, Southern Tohoku Proton Therapy Center, 7-172 Yatsuyamada, Koriyama, Fukushima 963-8052 Japan

**Keywords:** Stereotactic body radiotherapy, Radiation-induced rib fracture, Lung cancer, Fraction size, Fractionation

## Abstract

**Background:**

Radiation-induced rib fracture after stereotactic body radiotherapy (SBRT) for lung cancer has been recently reported. However, incidence of radiation-induced rib fracture after SBRT using moderate fraction sizes with a long-term follow-up time are not clarified. We examined incidence and risk factors of radiation-induced rib fracture after SBRT using moderate fraction sizes for the patients with peripherally located lung tumor.

**Methods:**

During 2003–2008, 41 patients with 42 lung tumors were treated with SBRT to 54–56 Gy in 9–7 fractions. The endpoint in the study was radiation-induced rib fracture detected by CT scan after the treatment. All ribs where the irradiated doses were more than 80% of prescribed dose were selected and contoured to build the dose-volume histograms (DVHs). Comparisons of the several factors obtained from the DVHs and the probabilities of rib fracture calculated by Kaplan-Meier method were performed in the study.

**Results:**

Median follow-up time was 68 months. Among 75 contoured ribs, 23 rib fractures were observed in 34% of the patients during 16–48 months after SBRT, however, no patients complained of chest wall pain. The 4-year probabilities of rib fracture for maximum dose of ribs (Dmax) more than and less than 54 Gy were 47.7% and 12.9% (p = 0.0184), and for fraction size of 6, 7 and 8 Gy were 19.5%, 31.2% and 55.7% (p = 0.0458), respectively. Other factors, such as D2cc, mean dose of ribs, V10–55, age, sex, and planning target volume were not significantly different.

**Conclusions:**

The doses and fractionations used in this study resulted in no clinically significant rib fractures for this population, but that higher Dmax and dose per fraction treatments resulted in an increase in asymptomatic grade 1 rib fractures.

## Background

Stereotactic body radiotherapy (SBRT) has been widely used for patients with inoperable primary and metastatic lung tumor. Historically, numerous clinical trials of SBRT for lung tumor with excellent primary control without severe toxicities have been reported [1-9]. Recently, however, frequencies of radiation-induced rib fracture, especially with peripherally located lung tumor treated with SBRT [10-12], have clearly become unexpectedly high compared to other methods of radiotherapy, such as tangential breast irradiation of breast conserving therapy [13].

Radiation-induced rib fracture is recognized as a late toxicity after external beam radiotherapy to the chest wall. From the radiobiological standpoint, larger fraction size is likely to increase the frequency of radiation-induced rib fracture [14]. However, fraction size for SBRT is relatively large; we have therefore started SBRT with moderate fraction size of 6 Gy since 2003 in order to avoid late toxicities and previously published our initial clinical experience of SBRT for lung tumors using 54 Gy in 9 fractions [15]. We have also started a dose escalation study of the fraction size by 1 Gy each from 6 Gy. However, some of the patients treated this way have developed rib fractures after SBRT. The purpose of this study is to investigate radiation-induced rib fracture, looking at incidence and risk factors for the patients with peripheral lung tumor treated with SBRT using moderate fraction sizes.

## Methods

This study was approved by the ethics committee of Hirosaki university school of medicine, and written informed consent was obtained from all patients.

### Patient and tumor characteristics

Between May 2003 and August 2008, 61 patients with medically inoperable lung tumor were treated with SBRT to 54–56 Gy in 9–7 fractions. The inclusion criteria for the current study were at least 15 months of radiographic follow-up, the tumor located within 2 cm of the chest wall, and patients with less than 10 mm of respiratory motion. Among the 61 patients, there were 6 patients excluded due to short follow-up time less than 15 months and 14 patients excluded due to tumor location. Therefore the subjects of this study included 41 patients (28 Stage IA lung cancers, 5 Stage IB lung cancers and 9 lung metastases). Among the 41 patients, one had two lung metastases, so 42 tumors were analyzed in this study. The patients and tumor characteristics are summarized in Table 1.

**Table 1:** **Patients and tumor characteristics**

**Patients (n = 41)**	
Age (y), mean and range	75 (45–86)
Sex (Male/Female)	29/12
Diagnosis	
Lung cancer (cT1N0M0)	28
Lung cancer (cT2N0M0)	5
Lung metastasis	9
Performance status (0/1/2)	23/16/2
Tumors (n = 42)	
Location (upper/middle/lower)	28/1/13
Location (anterior/lateral/posterior)	7/19/16
GTV (cm^3^), mean and range	7.6 (1–31)
PTV (cm^3^), mean and range	44.0 (11–134)

*Abbreviations:* GTV = gross tumor volume; PTV = planning target volume.

### Treatment procedure

All patients had raised both upper arms and were immobilized by a thermo-shell (ALCARE Co., Ltd., Tokyo, Japan) and a custom-made headrest using MoldCare (ALCARE Co., Ltd., Tokyo, Japan) during the treatment. After patient immobilization, respiratory movement of the tumor that was no more than 10 mm was confirmed with X-ray simulator (Toshiba Medical Systems Co., Ltd., Tokyo, Japan). Computed tomography (CT) was taken without breath holding by a CT-simulator (Aquilion, Toshiba Medical Systems Co., Ltd., Tokyo, Japan) with 2.0 mm thickness to recognize tumor localization and for dose calculation. A three-dimensional (3D) radiotherapy treatment-planning (RTP) machine (XiO, ver4.1.1, CMS Japan, Tokyo, Japan) was applied for the dose calculation. The outline of a target and normal tissues (total lung, spinal cord, vertebrae and or bronchus) were made for all patients. The target margins were defined as follows: clinical target volume (CTV) was equal to gross tumor volume (GTV); internal target volume (ITV) was CTV plus 5–10 mm margin according to respiratory movement of tumors confirmed with X-ray simulator; planning target volume (PTV) was CTV plus 5 mm margin in all directions according to set-up accuracy. A leaf margin was also taken 5 mm around the PTV. The patients in this study were treated before the introduction of 4D CT in our institution.

The dose calculation for the treatment was performed with Clarkson method by 3D-RTP corrected for inhomogeneity. The treatment was conducted on 42 lung tumors by fixed multiple non-coplanar conformal beams by a 10-MV standard linear accelerator with EXL-20TP (Mitsubishi Electric Co., Ltd., Tokyo, Japan). The beam arrangement consisted of 3 non-coplanar oblique anterior beams plus 2 coplanar oblique posterior beams plus 1 coplanar lateral beam.

Among the 41 patients meeting the inclusion criteria, fractionation schedules of SBRT were administered as follows: 54 Gy given in 9 fractions for initial 14 patients; 56 Gy given in 8 fractions for next 13 patients; 56 Gy given in 7 fractions for last 14 patients. The prescribed dose was defined at isocenter. The mean value of hot spots in the PTV was 56.26 Gy (range, 54.1–58.8 Gy). The hot spot in the PTV was within +5% of the isocentric dose (mean: 1.854%, range: 0.31 - 4.96%). The median overall treatment time was 12 days and ranged 9–22 days. The localization of the tumor was confirmed before every treatment by an electronic portal-imaging device (EPID), and EPID-based setup was performed on bony anatomy in this study.

### Contouring and dose-volume histograms

For the 41 patients meeting the inclusion criteria, all ribs where the irradiated doses were more than 80% of prescribed dose were contoured without margin for set-up movements. The dose calculation for the evaluation of radiation-induced rib fracture was performed with Clarkson method corrected for inhomogeneity. Each cumulative dose-volume histogram (cDVH) was exported to a file using a dose bin size of 0.2 Gy. The parameters of rib volume receiving 10–55 Gy (V10–55), dose at 2 cc of rib (D2cc), maximum dose of rib (Dmax), mean dose of rib (Dmean) and planning target volume (PTV) were analyzed using cDVHs. The dose calculation grid size for cDVHs was 3 mm, and the volume to define parameters of cDVHs was the whole scanning area including whole ribs in the study.

### Follow-up and statistics

**Figure 1:**
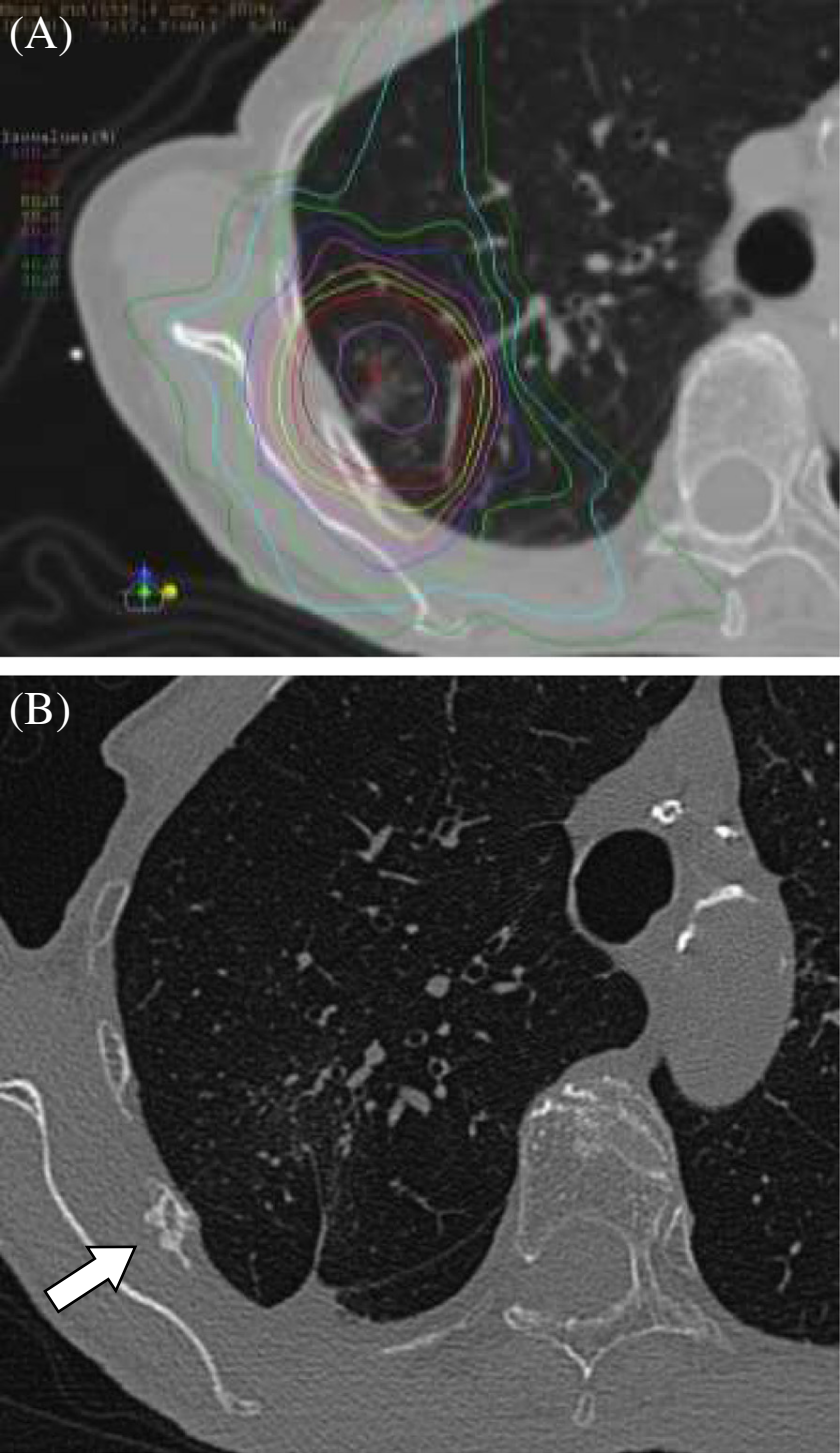
An 83-year-old woman with adenocarcinoma after SBRT with a total dose of 56 Gy given in 7 fractions. **(A):** Dosimetry overlaying CT with bone window shows the 95% iso-dose line on the right 5th rib. **(B):** Rib fracture was noted at 36 months after SBRT (arrow).

The endpoint in the current study was radiation-induced rib fracture, detected by CT scan at any time after the treatment; rib fractures according to tumor invasion were excluded in the current study. The end point was defined for each rib. Follow-up CT scans were obtained at 3–6 month intervals and were used to assess toxicity (Figure 1). All available CT scans were checked for rib fracture, not only of selected ribs but also excluded ribs. Toxicities were assessed according to the Common Terminology Criteria for Adverse Events Version 3.0 (CTCAE v3.0). The patients were also periodically monitored by medical examinations after the treatment.

All statistical analyses were performed with a commercial statistical software package (Dr. SPSS II for Windows, Version 11.0.1 J, SPSS Inc., Tokyo). The statistical significance of differences in mean values (V10–55, D2cc, Dmax, Dmean, and PTV) between two populations obtained by cDVHs was assessed with Student’s *t*-test. The actuarial curve was calculated with Kaplan-Meier method, according to the interval from the first date of the treatment, and differences in their distributions were evaluated using the log–rank test. Prognostic factors included in the analyses were age (≧75 vs. <75), sex (male vs. female), Dmax (≧54 Gy vs. < 54Gy), fraction size (6 Gy vs. 7 Gy vs. 8 Gy), PTV (≧50 cc vs. < 50 cc), tumor location (anterior vs. posterior vs. lateral: divided into three equal parts of the lung), rib location (1–4 vs. 5–8 vs. 9–12). Differences were regarded as statistically significant at *p* < 0.05.

## Results

### Incidence of radiation-induced rib fracture

Median follow-up time was 68 months, and ranged 16–130 months. For the 41 patients meeting the inclusion criteria, a total of 75 ribs were contoured. Among the 75 contoured ribs, 23 rib fractures were noted in 34% (14/41) of the patients during 16–48 months (median 30 months) after SBRT. No rib fracture was observed on excluded ribs in any patient in the study. Among the 14 patients with rib fractures, no one complained of chest wall pain, and the rib fractures healed without treatment; so the grade of fracture for the study patients was judged to be Grade1 according to CTCAE v3.0. Also, the remaining 27 patients without rib fractures, never complained of chest wall pain.

### Dose volume histogram

**Figure 2:**
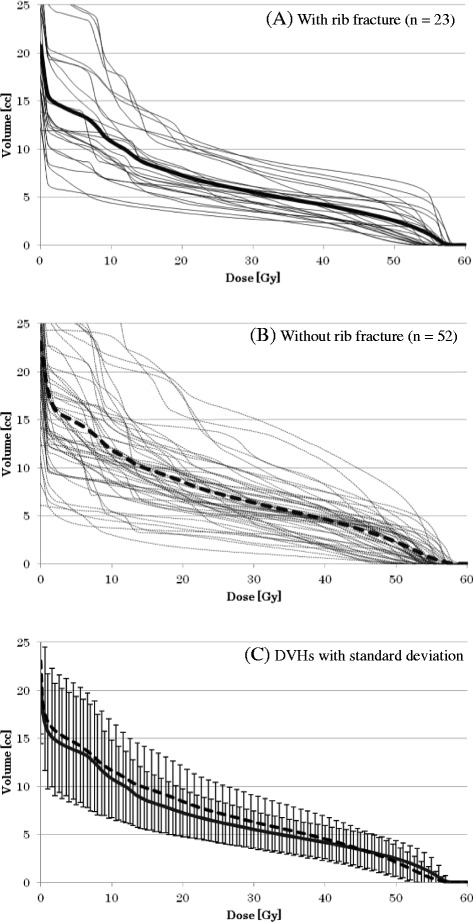
All cumulative DVHs for ribs. DVHs with fracture **(A)**, without fracture **(B)**, and DVHs with standard deviation **(C)** with fracture (solid curve) and without fracture (dotted curve).

The mean volume of the 75 contoured ribs was 22.5 cm^3^ (range: 6.0–39.5 cm^3^). The group with rib fracture had slightly larger volumes of the high dose level more than 50 Gy in comparison with the group without rib fracture on cDVHs. The cDVHs are shown in Figure 2.

The mean values of the variables (V10–55, D2cc, Dmax, Dmean, and PTV) according to with or without rib fracture are listed on Table 2. Only Dmax was a significant factor for the radiation-induced rib fracture (*p* = 0.012), whereas V10–55, D2cc, Dmean, and PTV were not significant.

**Table 2:** **Summary of mean values for categories with and without rib fracture**

**Categories**	**With fracture, n = 23**	**Without fracture, n = 52**	***p*** **value**
	**Mean value (range)**	**Mean value (range)**	
V10 (cc)	10.8 (4.4–18.6)	11.8 (3.0–28.7)	0.413
V20 (cc)	7.3 (3.4–11.6)	8.5 (1.7 - 18.2)	0.130
V30 (cc)	5.5 (2.7–9.1)	6.3 (1.1 - 15.1)	0.200
V40 (cc)	4.2 (1.9–7.9)	4.6 (0.6 - 11.7)	0.442
V50 (cc)	2.5 (0.3–6.7)	2.2 (0.0 - 8.0)	0.493
V53 (cc)	1.7 (0.01–6.0)	1.1 (0–5.0)	0.092
V54 (cc)	1.4 (0–5.6)	0.7 (0–4.5)	0.099
V55 (cc)	1.0 (0–4.8)	0.4 (0–3.6)	0.093
D2cc (Gy)	49.7 (38.6–56.8)	47.8 (16.7–56.4)	0.276
Dmax (Gy)	55.7 (53.4–58.4)	54.7 (50.1–58.8)	0.012
Dmean (Gy)	18.4 (12.6–33.7)	17.8 (11.1–29.2)	0.598
PTV (cc)	53.7 (14.8–133.7)	50.5 (13.8–133.7)	0.672

*Abbreviations:* Dmax = maximum dose of ribs; Dmean = mean dose of ribs; PTV = planning target volume.

### Probability of the radiation-induced rib fracture

**Figure 3:**
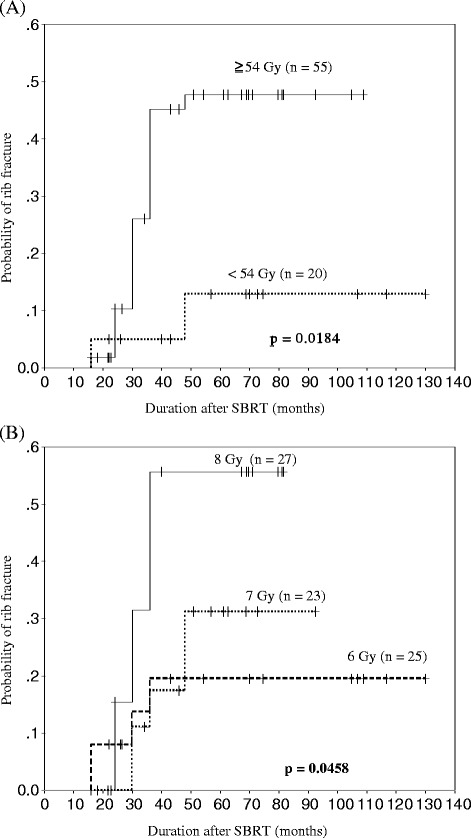
Cumulative incidence of radiation-induced rib fracture. Dmax more than and less than 54 Gy **(A)**, fraction size of 6, 7, and 8 Gy **(B)**.

Dmax of the rib and fraction size were significant factors for rib fracture on univariate analysis. The probability of the radiation-induced rib fracture at 4 years according to the Dmax of the rib less than and more than 54 Gy were 12.9% and 47.7%, respectively (*p* = 0.0184), which is shown in Figure 3A. The probability of the radiation-induced rib fracture at 4 years according to the fraction size of 6, 7 and 8 Gy were 19.5%, 31.2% and 55.7%, respectively (*p* = 0.0458), which is shown in Figure 3B. Other factors, such as age, sex, PTV, tumor and rib location were not significant on univariate analysis, which are summarized in Table 3.

**Table 3:** **Probabilities of radiation-induced rib fracture (n = 75)**

**Categories**	**n**	**4y-rates (%)**	**p values**
Age (y)			
≧75	47	37.5	NS
<75	28	40.0	
Sex			
Male	50	32.7	NS
Female	25	47.2	
Dmax (Gy)			
≧54	55	47.7	0.0184
<54	20	12.9	
Fraction size (Gy)			
6	25	19.5	0.0458
7	23	31.2	
8	27	55.7	
PTV (cc)			
≧50	28	41.2	NS
<50	47	35.5	
Tumor location			
Anterior	8	14.3	0.1097
Lateral	37	50.4	
Posterior	30	27.1	
Rib location			
1–4	40	31.0	0.0738
5–8	35	42.1	
9–12	6	75.0	

*Abbreviations:* Dmax = maximum dose of ribs; PTV = planning target volume; NS = not significant; HR = hazard ratio; CI = confidence interval.

No radiation-induced rib fracture was observed in the cases of fraction size with 6, 7, and 8 Gy for the value of Dmax of the ribs less than 53.6 Gy, 53.4 Gy, and 54.2 Gy, respectively. A cutoff value of the radiation-induced rib fracture for the Dmax of the rib was approximately 53 Gy, however, average values of Dmax with rib fracture were somehow related to the fraction size. They were 54.7, 55.5 and 56.1 Gy for 6, 7 and 8 Gy, respectively. Calculated biologically effective dose (BED8, α/β is assumed to be 8 Gy reported by Nambu et al. [16]) for the average values of Dmax in the patients with rib fracture, were 96.3, 103.6 and 112.3 Gy for the fraction size of 6, 7 and 8 Gy, respectively.

## Discussion

Radiation-induced rib fracture is one of the late radiation injuries not only for the patients with breast cancer treated with conventional postoperative radiotherapy but also for the patients with lung tumor treated with stereotactic body radiotherapy (SBRT). In our study, radiation-induced rib fracture was noted in 34% of the patients (14/41) during 16–48 months after SBRT. The incidence of rib fracture was significantly dependent upon Dmax and fraction size. The probability of the radiation-induced rib fracture at 4 years was approximately 50% for the Dmax more than 54 Gy and the fraction size of 8 Gy. The incidence of radiation-induced rib fracture was relatively high; however, no patients complained of chest wall pain and no rib fracture was noted for the rib with the Dmax less than 53 Gy.

The development of radiation-induced rib fracture with external beam radiotherapy is well documented. The incidence of radiation-induced rib fracture was 0.3–6% for patients with breast cancer. A retrospective analysis by Overgaard [14] examined the incidence of radiation-induced rib fracture detected by follow-up chest radiographs in 231 breast cancer patients treated with post mastectomy irradiation. The incidence of rib fracture was reported to be 19% for a large fraction size and 6% for a standard fraction size. Pierce et al. [13] reviewed 1,624 patients with early stage breast cancer treated by breast conserving therapy and reported the incidence of symptomatic rib fracture with or without x-ray confirmation to be 0.4–2.2%. A similar incidence of radiation-induced rib fracture detected by follow-up chest radiographs or CT has been reported in patients with non-small cell lung cancer treated with palliative radiotherapy by Quddus et al. [17].

When comparing these series with the literature regarding initial studies of SBRT, however, the incidence of radiation-induced pulmonary toxicity is well documented but the precise incidence of radiation-induced rib fracture is unknown. Uematsu et al. [1] reported a 5-year experience of SBRT using 50–60 Gy in 5–10 fractions with a median follow-up of 36 months for 50 patients, and reported that only one patient (2%) had a rib fracture detected by follow-up CT without requiring medical treatment. Onishi et al. [2] reported a clinical outcome in 245 subjects in a Japanese multi-institutional study of SBRT for stage I non-small cell lung cancer, and the incidence of rib fracture detected by follow-up CT was reported to be only 0.8% with a median follow-up of 24 months. In other early reports of SBRT, radiation-induced rib fracture has not been well documented, because the median follow-up time was relatively shorter and/or only central lesions were treated [3-6]. On the other hand, several recent studies of SBRT concerning radiation-induced rib fracture have been reported. Zimmermann et al. [7] reviewed 30 patients with stage I non-small cell lung cancer treated by SBRT with a total dose ranged from 24 to 37.5 Gy given in 3–5 fractions to the 60% isodose encompassing the planning target volume, and the incidence of rib fracture detected by follow-up CT was reported to be 3%. Similar incidence of radiation-induced rib fracture detected by follow-up CT with or without chest wall pain has been reported by Nyman et al. [8], and Fritz et al. [9].

However, incidence of rib fracture rises dramatically when treating lesions that are close to the chest wall. Voroney et al. [10] reported a series of 42 peripherally located lung lesions treated by SBRT with a total dose of 54–60 Gy in 3 fractions. The incidence of rib fracture was reported to be 21% (n = 9) with a median time to occurrence of 17 months, and rib fractures were symptomatic in 7 patients, whereas asymptomatic in 2 patients. Similar incidence of 16–24% for radiation-induced rib fracture detected by follow-up CT with or without chest wall pain after SBRT, and risk factors of dose and irradiated volume for the rib were reported by Pettersson et al. [11], Andolino et al. [12], and Asai et al. [18]. In addition, tumor location and patients’ factor are also associated with radiation-induced rib fracture. Nambu et al. [16] reported prevalence, degree of clinical symptoms, and risk factors for rib fracture detected by follow-up CT after SBRT with 177 patients, and found that small tumor-chest wall distance and female sex are risk factors for rib fracture. Kim et al. [19] reported risk factors for rib fracture detected by follow-up CT after SBRT with 118 patients with 2 year rib fracture rate of 42.4%, and concluded that female gender and lateral tumor location are risk factors for rib fracture. In this way, several factors associated with rib fracture must be considered, including dose, volume and site of irradiated rib, and patients’ characteristics. Further, the incidence of symptomatic rib fracture or asymptomatic rib fracture is also important issue. Nambu et al. [20] well discussed the incidence of rib fracture and chest wall pain for 177 primary lung cancer patients after SBRT. The incidence of symptomatic rib fracture, asymptomatic rib fracture, and chest wall pain without rib fracture were reported to be 7.9%, 15.3%, and 2.3%, respectively. In other words, the percentage of symptomatic rib fractures was 34.1% in their study with a total dose of 48–70 Gy in 4–10 fractions. Asai et al. [19] reported larger percentage of symptomatic rib fracture after SBRT with a total dose of 48 Gy in 4 fractions, and was 42.9%. On the other hand, no symptomatic rib fractures were observed in our study. Moderate fraction size of 6–8 Gy might be minimized symptomatic rib fracture.

In our study, even though moderate fraction size of 6–8 Gy used, radiation-induced rib fracture occur, and rib fracture is strongly correlated with Dmax and fraction size. The difference of the values of Dmax is only 1 Gy when comparing the Dmax with the DVH (Table 2), but a big difference is observed when comparing probability of rib fracture with Kaplan-Meier method (Figure 3A). The fraction size also had significant impact on the incidence of radiation-induced rib fracture. When comparing the fraction size with the same total dose (56 Gy per 8 fractions vs. 56 Gy per 7 fractions; for which fraction sizes were 7 Gy and 8 Gy, respectively), the probability of rib fracture according to the fraction size are significantly different, as shown in Figure 3B.

Based on these results, while normal tissue doses should always be minimized, it is not important to suppress rib dose within the range of doses used in this study. In addition, long-term follow-up is also necessary for the evaluation of radiation induced late bone damage, because the radiation-induced rib fractures occurred at 48 months after SBRT in our series. With a median follow-up period of 68 months in our series, fracture rate of 34% is higher than the crude rates of 3–5% quoted from initial studies of SBRT [7-9], but similar with recent studies for peripheral lung tumor [10-12,16,18,19]. Although the incidence of radiation-induced rib fracture is high, it does not necessarily require change in the treatment plan and/or prescribed dose, because the 14 patients with rib fracture did not complain of chest wall pain and the rib fracture healed without treatment. However, there is a report that obesity increases the risk of chest wall pain from SBRT [21,22], so it is necessary to pay attention in such patients.

## Conclusion

In conclusion, the doses and fractionations used in this study resulted in no clinically significant rib fractures for this population, but that higher Dmax and dose per fraction treatments resulted in an increase in asymptomatic grade 1 rib fractures.

## Abbreviations

SBRT: Stereotactic body radiotherapy
DVHs: Dose volume histograms
Dmax: Maximum dose of ribs
Dmean: Mean dose of ribs
GTV: Gross tumor volume
PTV: Planning target volume
NS: Not significant
HR: Hazard ratio
CI: Confidence interval
